# Comparative Transcriptome Analysis of Different *Actinidia arguta* Fruit Parts Reveals Difference of Light Response during Fruit Coloration

**DOI:** 10.3390/biology10070648

**Published:** 2021-07-11

**Authors:** Hailei Huang, Muhammad Abid, Miaomiao Lin, Ran Wang, Hong Gu, Yukuo Li, Xiujuan Qi

**Affiliations:** Zhengzhou Fruit Research Institute, Chinese Academy of Agricultural Sciences, Zhengzhou 450009, China; huanghailei2018@163.com (H.H.); 2018Y90100014@caas.cn (M.A.); linmiaomiao@caas.cn (M.L.); wangran@caas.cn (R.W.); guhong@caas.cn (H.G.)

**Keywords:** *Actinidia arguta*, coloration, photoresponse, transcriptome, AaMYB308like

## Abstract

**Simple Summary:**

Kiwifruit (*A. arguta*) color is one of the most important quality characters. Exploring the coloration mechanism is significant for the genetic improvement of color quality and the breeding of new germplasms. As a critical environmental factor, light plays a key role in fruit coloration. However, the effecting mechanism of light on *A. arguta* coloration remains unclear. In current research, different *A. arguta* parts with different treatments were performed high throughput RNA sequencing, based on which candidate genes and corresponding annotations were obtained. Finally, AaMYB308like was screened as an R2R3-MYB typed TF involved in light-inducible fruit coloration through the result analysis of bioinformatics and molecular biology experiments. Our study provides insights into the photoreponse mechanisms in *A. arguta* coloration.

**Abstract:**

Kiwifruit coloration is an important agronomic trait used to determine fruit quality, and light plays a vital role in the coloration process. The effect of light on fruit coloration has been studied in many species, but differences in the photoresponse of different fruit parts during fruit coloration is unclear in kiwifruit (*Actinidia arguta*). In this study, peel and core with bagging and non-bagging treatment at two stages were selected to perform high throughput RNA sequencing. A total of 100,417 unigenes (25,186 unigenes with length beyond 1000 bp) were obtained, of which 37,519 unigenes were annotated in functional databases. GO and KEGG enrichment results showed that ‘plant hormone signal transduction’ and ‘carbon metabolism’ were the key pathways in peel and core coloration, respectively. A total of 27 MYB-related TFs (transcription factors) were differentially expressed in peel and core. An R2R3-MYB typed TF, AaMYB308like, possibly served as a candidate objective, which played a vital role in light-inducible fruit coloration based on bioinformatics analysis. Transient overexpression of *AaMYB308like* suggested overexpression of *AaMYB308like* elevated transcription level of *NtCHI* in *Nicotiana tabacum* leaves. Integration of all these results imply that AaMYB308like might be served as a light-responsive transcription factor to regulate anthocyanin biosynthesis in *A. arguta*. Moreover, our study provided important insights into photoreponse mechanisms in *A. arguta* coloration.

## 1. Introduction

Kiwifruit belongs to Actinidiaceae, genus *Actinidia*, which possess rich germplasm resources, including 54 species and 21 varieties [1]. Some kiwifruit cultivars are red due to their high concentration of anthocyanins, metabolites with high antioxidant properties that contribute to improved vascular elasticity and protect against liver injury [2]. However, most kiwifruit cultivars are green, so there is a need to develop new red varieties given their potential benefit to human health. The whole-red *Actinidia arguta* (Sieb. et Zucc.) Planch. et Miq. is one of the new cultivated species in recent years. Due to the characteristics of the edible peel and special fruit with beautiful colors, *A. arguta* is popular among the consumers and in the sightseeing orchards in city suburbs [3]. However, during the actual process of production and cultivation, insufficient light resulting from orchard shading can cause abnormality in fruit coloration, and thus affect the marketing potential of fruits.

As an energy source of photosynthesis, light plays a pivotal role in plant growth and development. Additionally, light serves as an environment signal that participates in plant morphogenesis, which is commonly known photomorphogenesis [4,5,6]. Plants receive light signals through a series of receptors including phytochromes sensing red/far-red light, cryptochromes sensing blue/ultra-violet (UV)-A, phototropins and UVR8 sensing UV-B [7]. In fruit trees, some light response factors related to anthocyanin synthesis have been identified already. The apple MdHY5 was demonstrated to be an bZIP transcription factor that was induced by light. MdHY5 activated not only its own expression but key transcription factor MdMYB10 expression too, thus positively regulating anthocyanin biosynthesis [8]. Liu et al. (2019) identified another WRKY transcription factor MdWRKY11 that participated in anthocyanin accumulation by affecting MdMYB10, MdMYB11, MdUFGT, and photoresponse factor MdHY5 in red-fleshed apples, implying that some other novel genes that respond to light can also be involved in anthocyanin accumulation [9]. A bHLH transcription factor, FvbHLH9, was characterized in strawberry fruits. It was induced by light and functioned as a positive regulator involved in anthocyanin biosynthesis [10]. Therefore, the effect of light on fruit is received by a series of light response factors. So far, the related photoreponse factors have not been characterized in *A. arguta*.

Transcriptome refers to all the transcripts (mRNA, rRNA, tRNA, etc.) in a specific cell or tissue. The rapid development of high throughput sequencing technology provides a reliable and efficient platform for the systematic transcriptomic study of a specific biological trait [11,12]. Previously, numerous transcriptome analysis of bagging treatment in many species put emphasis on not only investigating the effect of light on anthocyanin biosynthesis but also finding the candidate light-responsive genes and pathways. Transcriptome analysis of peels from bagging-treated red Chinese sand pear provides knowledge about network interactions and reveals light-responsive pathway functions in anthocyanin biosynthesis [13]. Transcriptional level comparison of dark-/light-strawberry fruits by RNA-seq revealed the important role of light on anthocyanin synthesis, sugar accumulation and regulation of FvMYB10 [14]. Integrated results of transcriptome analysis of apple during light-induced anthocyanin accumulation and related biochemical index revealed fundamental insights into lncRNA involved in apple light-induced coloration [15]. Although transcriptome studies related to light-induced anthocyanin accumulation had been intensively reported, little transcriptome information about fruit coloration in *A. arguta* in response to light is available.

In this study, two fruit parts, peel and core with bagged/unbagged treatment at two developmental stages, were selected to perform RNA-seq. A total of 100,417 unigenes with an average length of 863.77 bp and N50 of 1600 bp were obtained from transcriptome analysis. In order to get DEGs (differentially expressed genes) and enrichment pathways that respond to light during fruit coloration, WP110 vs. TP110 and WX110 vs. TX110 served as the two main comparisons for deep exploration. Furthermore, a key photoreponse factor AaMYB308like was studied for functional validation. Our findings provided fundamental molecular mechanism underlying coloration in *A. arguta* in response to light.

## 2. Materials and Methods

### 2.1. Bagging Treatment, Sample Preparation, and Anthocyanin Measurement

**Figure 1:**
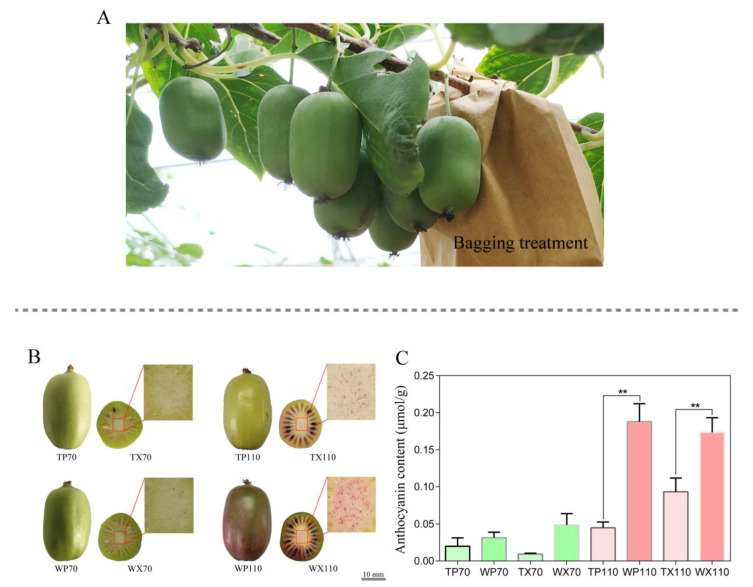
Sample treatment and preparation for RNA-seq. (**A**) Fruits were bagged using two-layer light-impermeable paper bags at one month after full bloom. (**B**) Peel and core were collected from bagged and unbagged fruit making a total of eight typed samples used for RNA-seq. (**C**) The anthocyanin content of these eight typed samples. Note: S1—fruit stage at 70 days after full bloom, S2—fruit stage at 110 days after full bloom, TP70—peel from bagged fruit at S1, TX70—core from bagged fruit at S1, TP110—peel from bagged fruit at S2, TX110—core from bagged fruit at S2, WP70—peel from unbagged fruit at S1, WX70—core from unbagged fruit at S1, WP110—peel from unbagged fruit at S2, WX110—core from unbagged fruit at S2. Data were analyzed with Student’s *t*-test (** *p* < 0.01).

Experimental design and sampling collection were performed on six independent *A. arguta* cv. ‘Tianyuanhong’ (‘TY’) vines. The experiment was repeated thrice with two vines in each biological replicate. Fruits at 30 days after full bloom (DAFB) were bagged using two-layer light-impermeable bags to ensure that the fruit in bags receives no light at all (Figure 1A). Untreated fruits receiving normal light were sampled at the same stages as the control. Sampling was carried out at two stages. The first sampling was done at 70 DAFB (S1) when the fruits were still green, and the second sampling was done at 110 DAFB (S2) when the fruit color was obviously different between bagged and unbagged fruits (Figure 1B). The peel and core were separated from fruits using a lab-used blade and were immediately frozen in liquid nitrogen and stored at −80 °C for subsequent use.

The measurement of anthocyanin content was conducted using previous methods [2], and all operations were performed for three replicates.

### 2.2. RNA Extraction, cDNA Synthesis, Library Construction, and Sequencing

RNA was extracted from sample using RNAprep Pure Plant Kit (DP441 TIANGEN, Beijing, China), after which the purity and concentration of RNA were tested by a NanoDrop 2000c spectrophotometer (Thermo Fisher Scientific, Waltham, MA, USA), and the integrity of RNA was tested using agarose gel electrophoresis, ensuring high qualification for the next procedure. The first cDNA strand was synthesized using random hexamer primer and M-MuLV Reverse Transcriptase (NEB, Rowley, NE, USA), and subsequently the second strand cDNA was obtained by DNA Polymerase I and RNase H (Thermo Fisher Scientific, Waltham, MA, USA). To get cDNA fragments of preferentially 240 bp in length, the library fragments were purified with AMPure XP system (Beckman Coulter, Beverly, MA, USA), and then 3 μL USER Enzyme (NEB, Rowley, NE, USA) was used with size-selected, adaptor-ligated cDNA at 37 °C for 15 min followed by 5 min at 95 °C before PCR. Afterwards, PCR was performed with Phusion High-Fidelity DNA polymerase (Roche, Basel, Switzerland), Universal PCR primers and Index (X) Primer. Finally, the PCR products were purified by AMPure XP system (A63880, Beckman Coulter, CA, USA) and library quality was assessed on the Agilent Bioanalyzer 2100 system. The prepared library was sequenced on an Illumina platform and paired-end reads were generated. There were three biological replicates performed for the RNA sequencing during whole RNA-seq analysis. In addition, all raw data has already been submitted to the public database NCBI Sequence Read Archive (SRA accession: PRJNA680442).

### 2.3. Quality Control and Gene Functional Annotation

Raw reads of fastq format were firstly processed through fastp software 0.21.0 (HaploX Biotechnology, Shenzhen, China) with specific parameters setting (-q 10 -u 50 -y -g -Y 10 -e 20 -l 100 -b 150 -B 150), during which the clean reads were obtained by removing adapter, poly-N, and low-quality reads. Meanwhile, Q20, Q30, GC-content, and sequence duplication level of clean reads were calculated to ensure downstream analyses based on high quality clean reads. The left files (read one file) from all libraries were pooled into one big left.fq file, and right files (read two files) into one big right.fq file. Transcriptome assembly was accomplished based on the left.fq and right.fq using Trinity with min_kmer_cov set to 2 by default and all other parameters set to default. Six databases, Nr (NCBI non-redundant protein sequences), Nt (NCBI non-redundant nucleotide sequences), Pfam (Protein family), KOG/COG (Clusters of Orthologous Group of proteins), Swiss-Prot (A manually annotated and reviewed protein sequence database), KO (KEGG Ortholog database), and GO (Gene Ontology), were used for gene functional annotation.

### 2.4. Quantification of Gene Expression and Differential Expression Analysis

FPKM (fragments per kilobase of transcript per million fragments mapped) was selected for estimation of gene expression quantification. DEseq was used for differential expression analysis [16,17]. DEseq provide statistical routines for determining differential expression in gene expression data using a model based on the negative binomial distribution. The resulting *p* value were adjusted using the Benjamini and Hochberg’s approach for controlling the false discovery rate. Genes with an adjusted *p*-value < 0.01 found by DEseq were assigned as differentially expressed.

### 2.5. GO and KEGG Enrichment Analysis

GO (gene ontology) enrichment analysis of the DEGs (differentially expressed genes) was implemented by the GOseq R packages based Wallenius non-central hyper-genometric distribution [18], which can adjust for gene length bias in DEGs. KEGG (Kyoto Encyclopedia of Genes and Genomes) is a database resource for understanding high-level functions and utilities of the biological system, such as the cell, the organism, and the ecosystem, from molecular-level information, especially large-scale molecular datasets generated by genome sequencing and other high throughput experimental technologies (http://www.genome.jp/kegg/, accessed on 1 January 2000). KOBAS software (3.0 version, Peking University, Beijing, China) was used for testing the statistical enrichment of differential expression genes in KEGG pathways [19].

### 2.6. Vector Construction and Transient Overexpression of AaMYB308like in N. tabacum

The CDS of *AaMYB308like* was amplified from *A. arguta* by specific primers 5′-GCTCTAGAATGGGGCGATCACCATGTTG-3′(forward) and 5′-CGGGATCCTCTACACAACCCTTGATTGC-3′(reverse) containing *Xba* I and *BamH* Ι restriction enzymatic sites. The PCR product was recombined with the plant binary expression vector pBI121 to form CaMV 35S:AaMYB308like-GFP. Empty pBI121 vector only with the GFP gene (35S:GFP) was used as control. Two constructs were introduced into *A. tumefaciens* strain EHA105 using a freeze-thaw method. *A. tumefaciens* strains were kept at 28 °C in LB medium with kanamycin antibiotic, re-suspended in infiltration buffer containing 10 mM MgCl_2_, 10 mM MES and 200 µM acetosyringone to OD_600_ of 0.6–1.0, and placed at room temperature for 2 h before infiltration.

The overexpression vector AaMYB308like-pBI121 constructed above was used for *A. tumefaciens*-mediated infiltration in tobacco leaves using a 1 mL needleless syringe. The empty vector pBI121-injected and non-treated leaves were both served as the control. Phenotypic observation and gene expression were assayed 7 days after transformation. Experimental operation was carried out on three biological repeats.

### 2.7. qRT-PCR Analysis

A total of 20 µL PCR mixture was used, including 5 µL ddH_2_O, 10 µL SYBR Green I master mix (Roche, Basel, Switzerland), 1 µL forward primer, 1 µL reverse primer, and 3 µL cDNA template. Real time PCR reaction was run on the LightCycler^®^ 480 system with a 96-well plate accompanied by the PCR procedure as follows: 95 °C for 5 min, followed by 45 cycles of 10 s at 95 °C, 20 s at 60 °C, and 20 s at 72 °C. The *Nicotiana tabacum* alpha-tubulin (*NtTubA1* gene) was selected as control for the transient overexpression qRT-PCR. The relative expression level of genes was estimated by the 2^−ΔΔCt^ method [20].

## 3. Results

### 3.1. Changes in Phenotype of Peel and Core between Bagging and Non-Bagging Treatment

The bagged and unbagged fruits were sampled at 70 DAFB and 110 DAFB. A total of eight types of samples from peel and core of fruits, namely TP70 (peel from bagged fruit at S1), TX70 (core from bagged fruit at S1), TP110 (peel from bagged fruit at S2), TX110 (core from bagged fruit at S2), WP70 (peel from unbagged fruit at S1), WX70 (core from unbagged fruit at S1), WP110 (peel from unbagged fruit at S2), and WX110 (core from unbagged fruit at S2) were used for RNA-seq analysis. The color of peel in TP70 and WP70 was still green (Figure 1B). However, the peel color of WP110 turned to red, while TP110 still remained green, which indicated that bagging treatment significantly suppressed peel coloration. TX110 core accumulated light red pigments compared to the WX110 core, which indicated that bagging failed to completely restrain the coloration process in core. The anthocyanin content of WP110 was significantly higher than that of TP110, meanwhile, the anthocyanin content of WX110 was significantly higher than that of TX110 (Figure 1C), which was consistent with the phenotypic presentation.

### 3.2. Transcriptome Assembly

Transcriptome analysis was performed on a total of 24 ‘TY’ samples, based on which 229,397 transcripts with N50 of 1856 and 100,417 unigenes with N50 of 1600 bp were obtained. The length distribution showed that with an increase of length of transcript and unigenes, their number and proportion gradually decreased. The specific distribution data for transcripts showed that 133,270 (58.09%) were shorter than 1000 bp, 55,806 (24.33%) were ranged between 1000 and 2000 bp, 40,321 (17.58%) were longer than 2000 bp, while data for unigenes presented that 75,231 (74.92%) were shorter than 1000 bp, 13,838 (13.78%) were ranged between 1000 and 2000 bp, 11,348 (11.30%) were longer than 2000 bp (Figure S1, Table 1). All in all, we obtained 100,417 unigenes, of which 25,186 (longer than 1000 bp) were used for further analysis.

### 3.3. Functional Annotation

To gain relevant functional information, all the unigenes obtained from RNA-seq were mapped to various functional databases. Among 100,417 unigenes, a total of 37,519 unigenes were annotated in databases including COG, GO, KEGG, KOG, Pfam, Swissprot, eggNOG, and NR (Table S1). Most of the unigenes could be annotated in NR and eggNOG databases, followed by Swissprot, Pfam, KOG, and GO. The distribution of unigenes among different databases was as follow: 34,561 were annotated in NR, 34,480 were annotated in eggNOG, 22,300 were annotated in Pfam, 22,123 were annotated in Swissprot, 21,560 were annotated in GO, 21,038 were annotated in KOG, 13,391 were annotated in KEGG and 10,167 were annotated in COG. The NR and eggNOG were top two among the databases, which include most of the annotated unigenes ranging from 300 to 1000 bp. The unigenes composed of more than 1000 bp followed a similar pattern (Table 2). The abovementioned results about annotated unigenes inferred that most of the unigenes could be successfully annotated in different databases, which could provide invaluable functional basic for further exploration of genes.

### 3.4. Expression Analysis of DEGs among Different Pairwise

The present study aimed to assess the differences in coloration process among different fruit parts. Therefore, a co-analysis was performed to find out common DEGs in six different comparisons for peel including WP110 vs. TP110, WP110 vs. WP70, WP110 vs. TP70, TP110 vs. WP70, TP110 vs. TP70 and WP70 vs. TP70, and six different comparisons for core including WX110 vs. TX110, WX110 vs. WX70, WX110 vs. TX70, TX110 vs. WX70, TX110 vs. TX70, and WX70 vs. TX70. A total of 4352, 6672, 6151, 3163, 2873, and 4436 DEGs were differentially expressed in WP110 vs. TP110, WP110 vs. WP70, WP110 vs. TP70, TP110 vs. WP70, TP110 vs. TP70, and WP70 vs. TP70, respectively (Table S2). A total of 5948, 7109, 7031, 2540, 2084, and 86 DEGs were differentially expressed in WX110 vs. TX110, WX110 vs. WX70, WX110 vs. TX70, TX110 vs. WX70, TX110 vs. TX70, and WX70 vs. TX70, respectively (Table S3). Co-analysis results showed that 205 DEGs were commonly expressed among six comparisons in peel (Figure S2a), while only 5 common DEGs were found in the core (Figure S2b). The presence of more common DEGs in the peels than that in the core indicated its significantly stronger response to light during fruit coloration, which was consistent with the phenotypic results (Figure 1B). The largest phenotypic difference occurred in 110 DAFB based on Figure 1B,C, so the DEGs were analyzed using volcano plot presence. A total of 2424 and 1928 transcripts were up-regulated and down-regulated expression in WP110 vs. TP110, respectively (Figure S2c, Table S4). A total of 3177 and 2771 transcripts were up-regulated and down-regulated expression in WX110 vs. TX110, respectively (Figure S2d, Table S5).

### 3.5. GO and KEGG Analysis of DEGs

**Figure 2:**
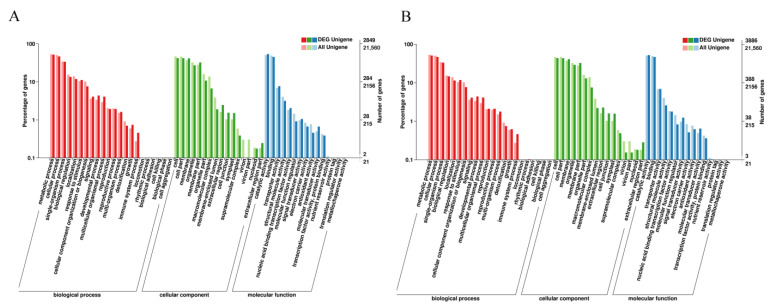
GO classification of DEGs from WP110 vs. TP110 and WX110 vs. TX110. (**A**) GO classification of DEG unigenes from WP110 vs. TP110. (**B**) GO classification of DEG unigenes from WX110 vs. TX110. The *x*-axis in (**A**,**B**) represents GO level-two terms belonging to three GO categories including biological process, cellular component, and molecular function. The *y*-axis in (**A**,**B**) represents the percentage and number of unigenes.

To find out the possible response of genes to light during peel and core coloration, the DEGs in WP110 vs. TP110 and WX110 vs. TX110 were selected to conduct GO and KEGG analysis. A total of 2849 DEGs were assigned to three GO terms including biological process, cellular component, and molecular function in WP110 vs. TP110. The ‘metabolic process’, ‘cellular process’, and ‘single-organism process’ were the top 3 level-two terms in biological process, followed by ‘biological regulation’, ‘localization’, and ‘response to stimulus’. The ‘cell’, ‘cell part’, and ‘membrane’ were the top 3 level-two terms in cellular component, followed by ‘organelle’, ‘membrane part’, and ‘organelle part’. The ‘catalytic activity’, ‘binding’, and ‘transporter activity’ were the top 3 level-two terms in molecular function, followed by ‘structural molecule activity’, ‘nucleic acid binding transcription factor activity’, and ‘molecular function regulation’ (Figure 2A). In order to obtain candidate genes involved in biological pathway, all unigenes were used to conduct KEGG analysis. A total of 883 DEGs were involved in 121 KEGG pathways in WP110 vs. TP110. Among these, 72, 71, and 70 DEGs were classified into ‘plant hormone signal transduction’, ‘ribosome’, and ‘biosynthesis of amino acids’, respectively, followed by 52 DEGs in ‘starch and sucrose metabolism’, 48 DEGs in ‘protein processing in endoplasmic reticulum’, and 48 DEGs in ‘carbon metabolism’ (Figure S3a, Table S6). These results suggested the possible involvement of the abovementioned pathways during peel coloration in response to light. For WX110 vs. TX110, there were 3886 DEGs assigned to three GO terms including biological process, cellular component, and molecular function. The specific level-two terms showed similar a distributed rule with that in WP110 vs. TP110 (Figure 2B). A total of 1225 DEGs were involved in 124 KEGG pathways in WX110 vs. TX110, among which 89, 83, and 83 DEGs were classified into ‘biosynthesis of amino acids’, ‘carbon metabolism’, and ‘ribosome’, respectively, followed by 80 DEGs in ‘starch and sucrose metabolism’, 76 DEGs in ‘protein processing in endoplasmic reticulum’, and 75 DEGs in ‘plant hormone signal transduction’, indicating that these pathways played a key role in core coloration (Figure S3b, Table S6). The genes and main pathways were different in peel and core during fruit coloration, which indicated the presence of different response mechanism in peel and core to light. In addition, to find out the interesting genes involved in anthocyanin biosynthesis, hormone signal transduction and carbon metabolism of *A. arguta* fruit subjected to light treatment, 12 possible genes were screened from transcriptome data (Table S7, Figure S4), which would provide important reference for future study.

### 3.6. The Possible MYB-Related Genes Controlling Coloration of Different Fruit Parts

**Figure 3:**
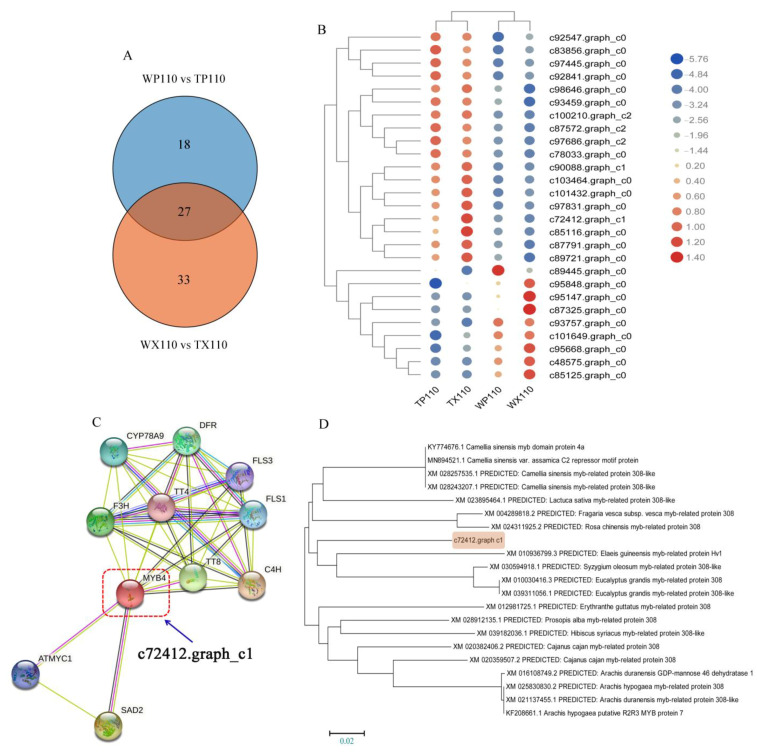
The possible MYB-related genes involved in light-inducible fruit coloration. (**A**) Venn diagram of MYB-typed DEGs between WP110 vs. TP110 and WX110 vs. TX110. (**B**) The transcription level of 27 MYB-typed DEGs. (**C**) The analysis of functional protein association networks in *Arabidopsis* for MYB4 as the closest ortholog available of c72412.graph_c1. (**D**) Phylogeny analysis of c72412.graph c1 and its top 20 homologous genes in NCBI blast. The way of phylogenetic tree construction is Construct/Test Neighbor-Joining Tree in MEGA 6.0 software (Version 6.0, Mega Limited, Auckland, New Zealand). The orange shadow mutates the transcript c72412.graph c1. The setting bar for genetic distance is 0.02.

As one of the largest families in plant transcription factors, MYB typed TFs play a vital role in plant growth and development as well as fruit coloration. To find out if the candidate MYB-typed TFs, WP110 vs. TP110 and WX110 vs. TX110 continued to be served as the excavating point for further exploration. There was a total of 45 and 60 MYB-related DEGs in WP110 vs. TP110, and WX110 vs. TX110, respectively. Besides, a total of 27 common DEGs were found between each other (Figure 3A, Table S8). There were two kinds of different expression patterns for these 27 DEGs, 18 of which were up-regulated after bagging treatment, while the other 9 DEGs presented decreasing trend (Figure 3B), indicating that these MYB-related DEGs could respond to light. To find out which gene was involved in anthocyanin biosynthesis, the analysis of functional protein association networks was performed in online STRING database (https://string-db.org/cgi, accessed on 2 November 2010). Interestingly, among 27 DEGs, only one gene, c72412.graph c1 (the closest ortholog gene of MYB4 in *Arabidopsis*), interacted with anthocyanin-related genes (Figure 3C). To explore the specific information about c72412.graph c1, NCBI-blast was conducted to search the top 20 homologous genes. The phylogenetic tree showed that c72412.graph c1 was very likely a *AaMYB308like* gene (Figure 3D), based on which we named c72412.graph c1 as *AaMYB308like*.

### 3.7. Transient Overexpression of AaMYB308like in Nicotiana tabacum

**Figure 4:**
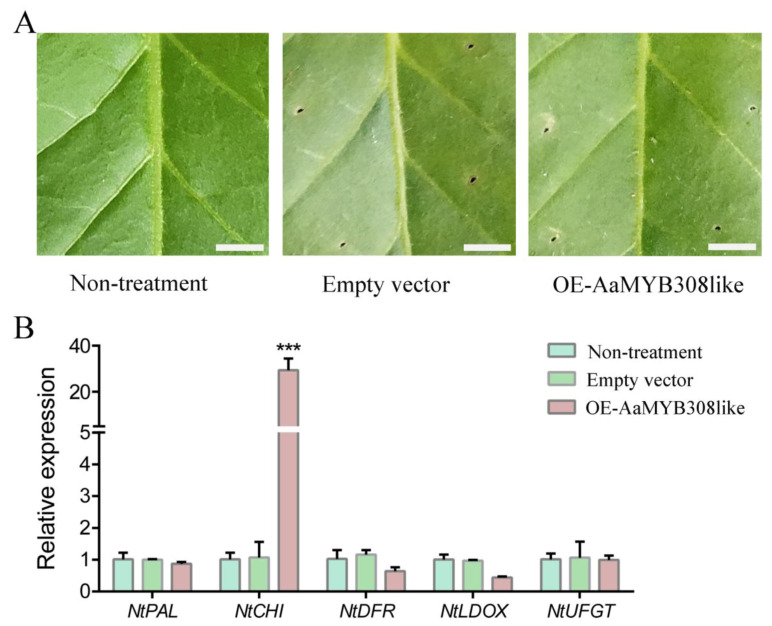
Transient overexpression of *AaMYB308like* in *Nicotiana tabacum* leaves. (**A**) Phenotype of *Nicotiana tabacum* leaves five days after *AaMYB308like* overexpression. Scale bars: 1 cm. (**B**) Relative expression level of structural genes involved in anthocyanin biosynthetic pathway. OE represents overexpression of *AaMYB308like*. Data were analyzed with Student’s *t*-test (*** *p* < 0.001).

In order to further investigate the specific role of AaMYB308like in anthocyanin regulation, transient overexpression of AaMYB308like was carried out in *N. tabacum* leaves by *Agrobacterium*-mediated transformation. Phenotypic observation of tobacco after infiltration showed that the leaves of transiently over-expressed AaMYB308like showed no obvious changes compared with the control with the empty vector or no treatment (Figure 4A). However, the transcription levels of structural genes including *NtPAL*, *NtCHI*, *NtDFR*, *NtLDOX,* and *NtUFGT* investigated in corresponding leaves showed a different expression level. In over-expressed tobacco leaves, the over-expression of AaMYB308like significantly up-regulated expression levels of *NtCHI*, but did not change the expression of other genes (Figure 4B), which suggested that overexpression of AaMYB308like could activate *NtCHI* expression in tobacco.

## 4. Discussion

Fruit color is an important agronomic trait and is responsible for market value. As ready-to-eat fruit, *A. arguta* coloration plays a key role in ensuring fruit quality and attracting consumers. Light is a key environmental factor that mediates fruit coloration by regulating related genes [21,22]. To gain molecular information of the photoresponse mechanism of different parts of *A. arguta*, we performed high throughput RNA-seq for a total of 24 samples. Finally, we obtained 100,417 unigenes with an average length of 863.77 bp and N50 of 1600 bp, of which 37,519 unigenes were annotated in functional databases (Tabel 2). Co-analysis presented 205 DEGs in peel and 5 DEGs in core (Figure S2), which indicated a significantly stronger response of peel than that of core to light during fruit coloration. To find out the key differences between peel and core coloration process, WP110 vs. TP110 and WX110 vs. TX110 were selected as two main comparisons for further analysis. The 2849 and 3886 DEGs were classified into GO terms in WP110 vs. TP110 and WX110 vs. TX110, respectively. Similarly, 883 and 1225 DEGs were assigned into KEGG pathways in WP110 vs. TP110 and WX110 vs. TX110, respectively (Figure 2, Figure S3). Different DEGs involved in GO and KEGG suggested that peel and core had a different photoresponse mechanism during fruit coloration. Based on the KEGG enrichment results, we can conclude that ‘plant hormone signal transduction’ is the most important pathway in peel coloration, while ‘carbon metabolism’ is the most important pathway in core coloration. As the outermost part of *A. arguta*, the peel coloration is an important aspect for fruit appearance and market value.

To further explore the specific genes involved in peel coloration, a light responsive transcription factor AaMYB308like was cloned from *A. arguta* peel. In order to further confirm its function during the coloring process, the AaMYB308like-PBI121 expression vector was constructed and transiently transformed into *N. tabacum* leaves. The results showed that transient overexpression of AaMYB308like in *N. tabacum* leaves significantly up-regulated *NtCHI* (EBG) expression, which suggests the possible involvement of AaMYB308like in anthocyanin biosynthesis. However, there were no changes of color and anthocyanin accumulation, which indicated the inadequacy of AaMYB308like in activating the whole anthocyanin biosynthetic pathway (Figure 4). Our results were at part with a previous study, where transient transformation of AcMYB5-1/5-2/A1-1 cloned from *A. chinenses* in *N. benthamiana* leaves showed no change in leaf color and accumulation of anthocyanin [23].

To perform the homology analysis between AaMYB308like identified above and other anthocyanin-related MYB typed transcription factors, the MYB TFs sequences in model plants, such as *Arabidopsis thaliana* and *Solanum lycopersicum*, and several fruit plants including *Malus domestica*, *Citrus sinensis*, *Prunus persica*, *Pyrus pyrifolia*, *Vitis vinifera*, *Fragaria ananassa*, *Fragaria vesca*, *Actinidia chinensis,* and *Actinidia arguta* were collected from NCBI database. A total of 18 MYB TFs were selected for the sequence and homology analysis. The homology of AaMYB308like and these 18 MYB TFs were maintained at a middle level with the identity ranging from 45.68% to 69.83%, which indicates that AaMYB308like was indeed a MYB typed TF and played a regulating role in anthocyanin biosynthesis (Table 3). MdMYB1 was a key TF regulating anthocyanin biosynthesis in apple (Malus domestica) by not only activating expression of downstream structural genes, but also interacting with other TFs including MdERF3 (ETHYLENE RESPONSE FACTOR 3) and MdEIL1 (ETHYLENE INSENSITIVE 3-LIKE 1) to mediate ethylene responding fruit coloration. Additionally, as a light response factor, MdMYB1 was also served as a light response factor participating in the light-mediated anthocyanin regulation [24,25]. In our study, AaMYB308like was screened as a candidate light response factor to regulate anthocyanin biosynthesis, showing the possible consistency with MdMYB1. However, whether AaMYB308like works in the same way with MdMYB1 involved in fruit coloration still needs further confirmation. Besides in other species, MYB transcription factors in kiwifruit have also been found to be involved in anthocyanin regulation. AcMYB75 was identified to be an R2R3-MYB TF to regulate anthocyanin accumulation by activating the promoter of *AcANS* in *A. chinensis* [26]. Whether AaMYB308like regulates anthocyanin biosynthesis in a similar way needs to be confirmed. Overall, the candidate AaMYB308like TF could be used as the key factor to participate in light-mediated anthocyanin regulation, while the specific regulatory mechanism needs to be further explored.

## 5. Conclusions

In the present study, we revealed the molecular differences in the coloration process between *A. arguta* peel and core. The ‘plant hormone signal transduction’ was the key pathway during peel coloring process, while the ‘carbon metabolism’ was the key pathway in core coloring process. Additionally, transient overexpression of AaMYB308like in *N. tabacum* confirmed the active role of photoresponse factor AaMYB308like in anthocyanin biosynthesis. This study will provide significant molecular resources for understanding the intricate mechanisms and pathways involved in anthocyanin biosynthesis. Furthermore, this study will help the researchers to improve the fruit appearance.

## Figures and Tables

**Table 1:** Assembly summary of 24 ‘TY’ transcriptome samples.

Transcript Length	Total Number	Percentage	Unigene Length	Total Number	Percentage
200–300	36,864	16.07%	200–300	28,565	28.45%
300–500	42,911	18.71%	300–500	26,507	26.40%
500–1000	53,495	23.32%	500–1000	20,159	20.08%
1000–2000	55,806	24.33%	1000–2000	13,838	13.78%
>2000	40,321	17.58%	>2000	11,348	11.30%
Total number	229,397		Total number	100,471	
Total length	267,448,645		Total length	86,736,802	
N50 length	1856		N50 length	1600	
Mean length	1165.88		Mean length	863.77	

**Table 2:** The general overview of the number of annotated unigenes in databases including COG, KEGG, KOG, GO, Swissprot, Pfam, eggNOG, and NR.

Database	Number of Annotated Unigenes	“Length” (300–1000)	“Length” (>1000)
COG	10,167	2519	6358
KEGG	13,391	4343	7123
KOG	21,038	6921	10,791
GO	21,560	6918	11,001
Swissprot	22,123	6590	13,062
Pfam	22,300	6416	13,379
eggNOG	34,480	11,576	17,083
NR	34,561	11,396	17,548
All_Annotated	37,519	12,916	17,787

**Table 3:** Information for the anthocyanin-related MYB transcription factors in other species.

Gene Name	Species	NCBI GenBank Accession	Length/bp	Identity with AaMYB308like	References
*AtPAP1*	*Arabidopsis thaliana*	NM_104541.4	747	61.76%	[27]
*AtMYB113*	*Arabidopsis thaliana*	NM_105308.2	741	56.52%	[27]
*SlMYB75*	*Solanum lycopersicum*	NM_001279063.2	828	59.29%	[28,29,30,31,32]
*MdMYB1*	*Malus domestica*	NM_001301116.1	1239	46.85%	[33,34,35,36,37]
*MdMYB10*	*Malus domestica*	EU518249.2	732	59.43%	[38]
*CsRUBY*	*Citrus sinensis*	NM_001288889.1	789	45.68%	[39,40]
*PpMYB10*	*Prunus persica*	EU155160.1	675	64.29%	[41]
*PyMYB10*	*Pyrus pyrifolia*	GU253310.1	735	62.00%	[42]
*VvMYBA1*	*Vitis vinifera*	B242302.1	753	50.00%	[43]
*FaMYB10-1*	*Fragaria ananassa*	MG456859.1	702	65.69%	[44]
*FaMYB10-2*	*Fragaria ananassa*	MG456860.1	540	65.69%	[44]
*FvMYB10*	*Fragaria vesca*	EU155163.1	708	65.69%	[44]
*AcMYB10*	*Actinidia chinensis*	MG581953.1	666	64.76%	[45]
*AcMYB75*	*Actinidia chinensis*	KX349735.1	666	64.76%	[26]
*AcMYB110*	*Actinidia chinensis*	KF311107.1	729	57.03%	[46]
*AcMYBF110*	*Actinidia chinensis*	MH370827.1	666	64.76%	[47]
*AcMYB123*	*Actinidia chinensis*	MH643775.1	801	68.50%	[48]
*AaMYBC1*	*Actinidia arguta*	MN249175.1	798	69.83%	[3]

## Data Availability

Not applicable.

## Supplementary Materials

The following are available online at https://www.mdpi.com/article/10.3390/biology10070648/s1, Figure S1: Distribution of unigenes and transcripts, Figure S2: Venn diagram and volcano plot of DEGs, Figure S3: KEGG enrichment of DEGs from WP110 vs. TP110 and WX110 vs. TX110, Figure S4: The expression patterns of 12 possible genes were involved in three KEGG pathways including anthocyanin biosynthesis, hormone signal transduction, and carbon metabolism, Table S1: The annotation information of unigenes in databases, Table S2: Information for six comparisons in peel, Table S3: Information for six comparisons in core, Table S4: The up/down-regulated transcripts in WP110 vs TP110, Table S5: The up/down-regulated transcripts in WX110 vs TX110, Table S6: KEGG enrichment information of DEGs in WP110 vs TP110 and WX110 vs TX110, Table S7: The specific annotation information of a representative group of DEGs related to three main pathways including ‘anthocyanin biosynthesis’, ‘plant hormone signal transduction’ and ‘carbon metabolism’ involved in light-dependent fruit (peel and core) coloration, Table S8: MYB-typed DEGs in WP110 vs TP110 and WX110 vs TX110.
