# Supplementary material for: Comparative Transcriptome Analysis of Different Actinidia arguta Fruit Parts Reveals Difference of Light Response during Fruit Coloration
Source: Biology (Basel). 2021 Jul 11;10(7):648. doi: 10.3390/biology10070648 (PMC8301191; doi:10.3390/biology10070648)
Supplement: Supplementary file 1 [file biology-10-00648-s001.zip › Fig S1-S4.pdf]

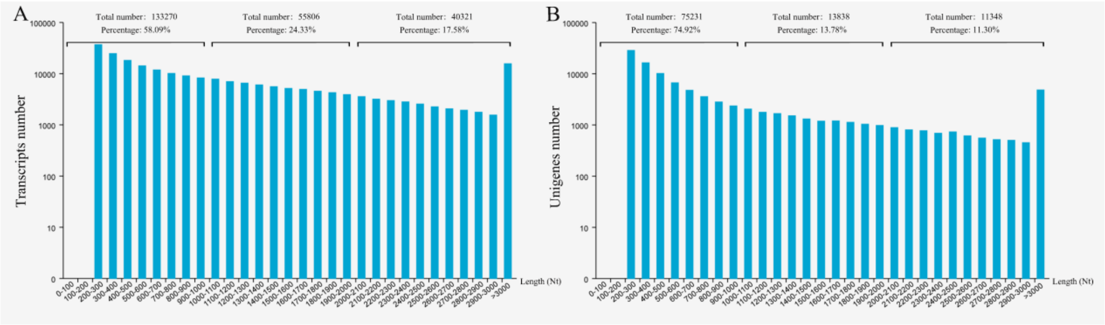

Supplementary Figure S1. Distribution of unigenes and transcripts. (A) Length distribution of transcript. The x-axis represents different length interval of transcript, while y-axis represents transcript number. (B) Length distribution of unigene. The x-axis represents different length interval of unigene, whereas y-axis represents unigene number.

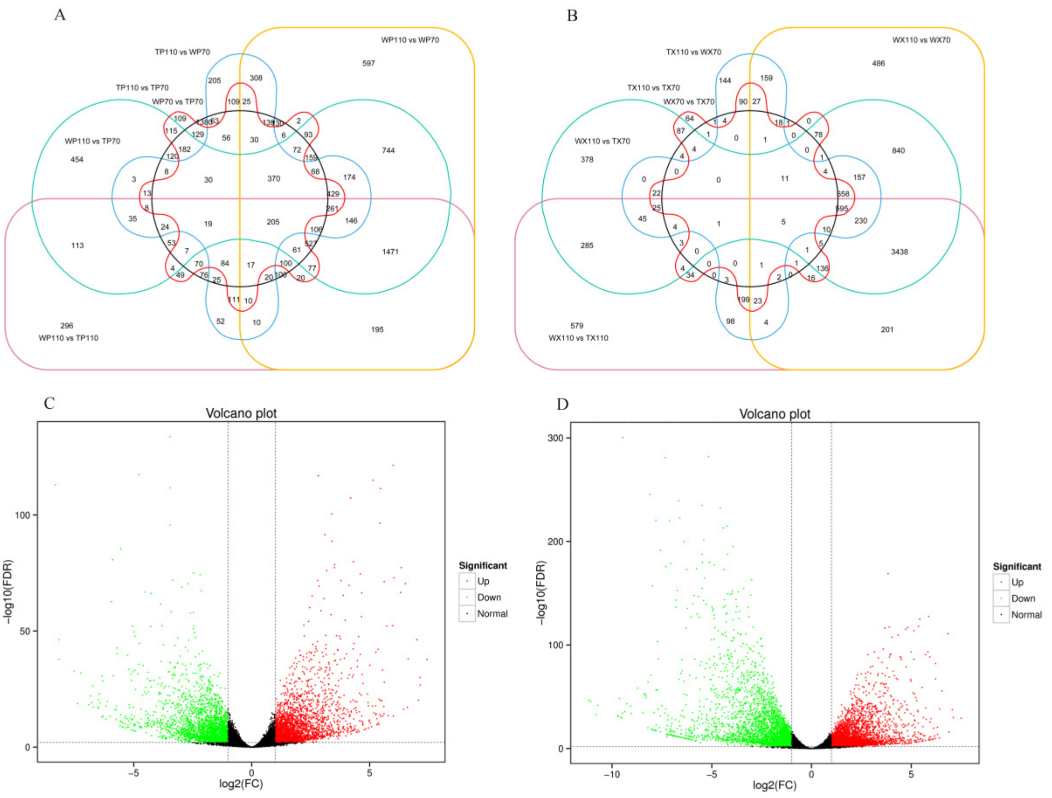

Supplementary Figure S2. Venn diagram and volcano plot of DEGs. (A) Venn diagram of DEGs among WP110 vs TP110, WP110 vs WP70, WP110 vs TP70, TP110 vs WP70, TP110 vs TP70 and WP70 vs TP70. (B) Venn diagram of DEGs among WX110 vs TX110, WX110 vs WX70, WX110 vs TX70, TX110 vs WX70, TX110 vs TX70 and WX70 vs TX70. (C) Volcano plot of DEGs in WP110 vs TP110 comparison. (D) Volcano plot of DEGs in WX110 vs TX110. **Note:** TP70 - peel from bagged fruit at S1, TX70 - core from bagged fruit at S1, TP110 - peel from bagged fruit at S2, TX110 - core from bagged fruit at S2; WP70 - peel from unbagged fruit at S1, WX70 - core from unbagged fruit at S1, WP110 - peel from unbagged fruit at S2, WX110 - core from unbagged fruit at S2.

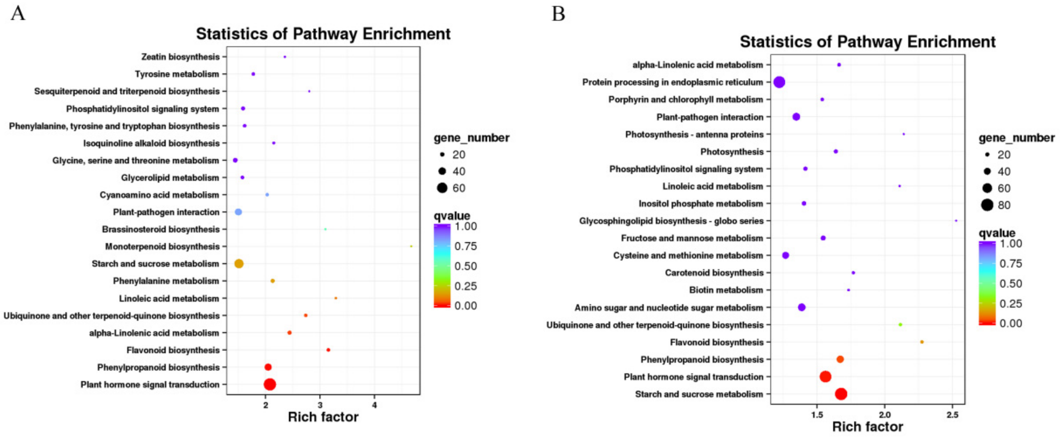

Supplementary Figure S3. KEGG enrichment of DEGs from WP110 vs TP110 and WX110 vs TX110. (A) KEGG pathway enrichment of DEG unigenes from WP110 vs TP110. (B) KEGG pathway enrichment of DEG unigenes from WX110 vs TX110. The x-axis in (A) and (B) represents rich factor and the y-axis represents specific KEGG terms.

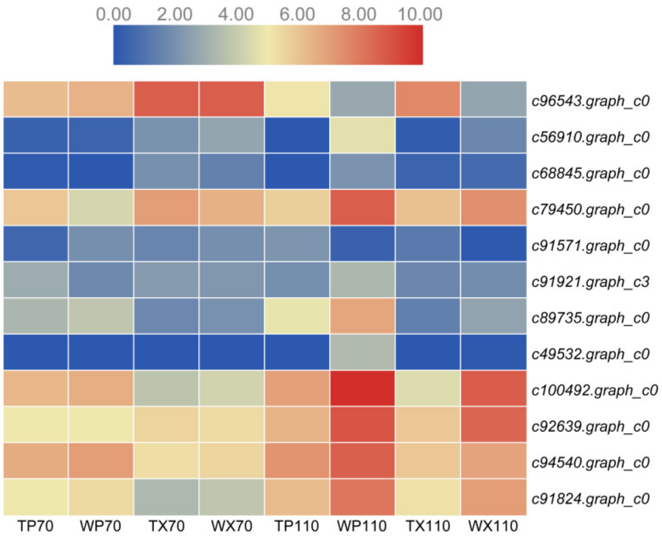

Supplementary Figure S4. The expression patterns of 12 possible genes involve in three KEGG pathways including anthocyanin biosynthesis, hormone signal transduction and carbon metabolism.
