# Supplementary material for: Comparative Transcriptome Analysis of Different Actinidia arguta Fruit Parts Reveals Difference of Light Response during Fruit Coloration
Source: Biology (Basel). 2021 Jul 11;10(7):648. doi: 10.3390/biology10070648 (PMC8301191; doi:10.3390/biology10070648)
Supplement: Supplementary file 1 [file biology-10-00648-s001.zip › Table S7.pdf]

Table S7. The specific annotation information of a representative group of DEGs related to three main pathways including 'anthocyanin biosynthesis', 'plant hormone signal transduction' and 'carbon metabolism' involved in light-dependent fruit (peel and core) coloration.

Gene ID	KEGG pathways	Nr annotation
c100492.graph_c0	“anthocyanin biosynthesis”	chalcone synthase
c92639.graph_c0	“anthocyanin biosynthesis”	flavanone 3-hydroxylase
c94540.graph_c0	“anthocyanin biosynthesis”	chalcone isomerase
c91824.graph_c0	“anthocyanin biosynthesis”	flavonoid 3-0-galactosyltransferase
c96543.graph_c0	“plant hormone signal transduction”	auxin early response protein AUX/IAA27
c56910.graph_c0	“plant hormone signal transduction”	auxin-induced protein 22D-like
c68845.graph_c0	“plant hormone signal transduction”	auxin-induced protein 22A-like
c79450.graph_c0	“plant hormone signal transduction”	hypothetical protein POPTR_010G078400v3
c91571.graph_c0	“carbon metabolism”	8-hydroxygeraniol oxidoreductase
c91921.graph_c3	“carbon metabolism”	PREDICTED: alcohol dehydrogenase-like 7
c89735.graph_c0	“carbon metabolism”	PREDICTED: cysteine synthase-like
c49532.graph_c0	“carbon metabolism”	Ketose-bisphosphate aldolase class-2
